# Transcriptome Analysis of *Dastarcus helophoroides* (Coleoptera: Bothrideridae) Using Illumina HiSeq Sequencing

**DOI:** 10.1371/journal.pone.0100673

**Published:** 2014-06-30

**Authors:** Wei Zhang, Wang Song, Zhengqing Zhang, Haidong Wang, Miaomiao Yang, Ruijian Guo, Menglou Li

**Affiliations:** 1 College of Forestry, Northwest A&F University, Yangling, Shaanxi, People's Republic of China; 2 Laboratory of Agrozoology, Department of Crop Protection, Faculty of Bioscience Engineering, Ghent University, Ghent, Belgium; 3 Shaanxi Institute of Zoology, Xi'an, Shaanxi, People's Republic of China; University of Innsbruck, Austria

## Abstract

**Background:**

*Dastarcus helophoroides* is known as the most valuable natural enemy insect against many large-body longhorned beetles. The molecular mechanism of its long lifespan and reproduction makes it a unique resource for genomic research. However, molecular biological studies on this parasitic beetle are scarce, and genomic information for *D. helophoroides* is not currently available. Thus, transcriptome information for this species is an important resource that is required for a better understanding of the molecular mechanisms of *D. helophoroides*. In this study, we obtained transcriptome information of *D. helophoroides* using high-throughput RNA sequencing.

**Results:**

Using Illumina HiSeq 2000 sequencing, 27,543,746 clean reads corresponding to a total of 2.48 Gb nucleotides were obtained from a single run. These reads were assembled into 42,810 unigenes with a mean length of 683 bp. Using a sequence similarity search against the five public databases (NR, Swiss-Prot, GO, COG, KEGG) with a cut-off E-value of 10^−5^ using Blastx, a total of 31,293 unigenes were annotated with gene description, gene ontology terms, or metabolic pathways.

**Conclusions:**

To the best of our knowledge, this is the first study on the transcriptome information of *D. helophoroides*. The transcriptome data presented in this study provide comprehensive information for future studies in *D. helophoroides*, particularly for functional genomic studies in this parasitic beetle.

## Introduction

*Dastarcus helophoroides* (Faimaire) (Coleoptera: Bothrideridae) is the most effective natural enemy of many large-body longhorned beetles, including *Anoplophora glabripennis*, *Monochamus alternatus*, *Batocera horsfieldi*, and *Massicus raddei* [1]. *D. helophoroides* larvae are ecto-parasitoids of late instar larvae, pupae, and young adults of longhorned beetles, which makes it a potential biological control agent for pest management [2]–[4]. Longhorned beetles (Coleoptera: Cerambycidae) are major pests of forestry production, crop cultivation, and construction timbers, some of which are vectors of pine wood nematodes [5]–[6]. Longhorned beetles are distributed worldwide; however, the parasitic beetles are mainly distributed in both China and Japan, and investigations have been performed in these two regions [7]–[9]. Over the past few decades, *D. helophoroides* have been intensively studied for their importance in the biological control of longhorned beetles. In addition to being the most effective natural enemy of many longhorned beetles, *D. helophoroides* are also important due to their long lifespan.

Most adult insects have shorter lifespans, except some advanced social insects. For example, the maximal lifespan of ant queens, termite kings and queens, and honeybee queens is 30 years [10], 12 years [11], and 8 years [12], respectively. However, most male adults have shorter lifespans than do the females. Under laboratory conditions, *D. helophoroides* can live for more than 8 years with continued sexual reproduction [13]. This feature provides a unique resource for molecular and physiological studies of development and reproduction. Although the morphology and physiology of *D. helophoroides* have been widely reported, its molecular mechanism of development and reproduction remain unknown [14]. As of September 24, 2013, only 48 *D. helophoroides* nucleotide sequences and 30 protein sequences have been deposited in the NCBI database. These data are far from sufficient, and most of the important genes related to development and reproduction are still unknown. Because genomic information for *D. helophoroides* is not currently available, detailed transcriptome data of *D. helophoroides* are expected to improve our understanding of their molecular mechanisms of development and reproduction.

The emergence of next generation sequencing technologies have dramatically accelerated genome-wide studies of transcriptomes and have been widely used to explore gene structure and gene expression, even without a genome reference [14]–[19]. RNA-seq has enabled *de novo* transcriptome sequencing with the development of short read sequencing technologies, such as the Roche 454, SOLiD and Solexa/Illumina platforms for various purposes [20]–[21]. Illumina HiSeq RNA sequencing is a recently developed high-throughput sequencing method that has been shown to be a reliable and precise method to study genomic characteristics, including development, insecticide targets, detoxifying enzymes, metabolism and immune response, and tissue specificity [22]–[29]. The success of this research is dependent on the availability of deep and detailed transcriptome data of *D. helophoroides*, which is expected to improve our understanding of *D. helophoroides* at the molecular level.

In this study, we used Illumina HiSeq RNA sequencing technology for *de novo* transcriptome analysis. We constructed a library using adult *D. helophoroides*. Approximately 27.5 million reads with a total of 2.5 billion nucleotides were assembled into 42,810 unigenes, of which 30,103 (70.32%) unigenes matched known proteins in a BLAST search of the NCBI database. These assembled and annotated transcriptome sequences extend the genomic resources available for researchers studying *D. helophoroides* and may provide a fast approach to identify the genes involved in development and reproduction.

## Results

### *De novo* sequence assembly of the transcriptome

**Figure 1:**
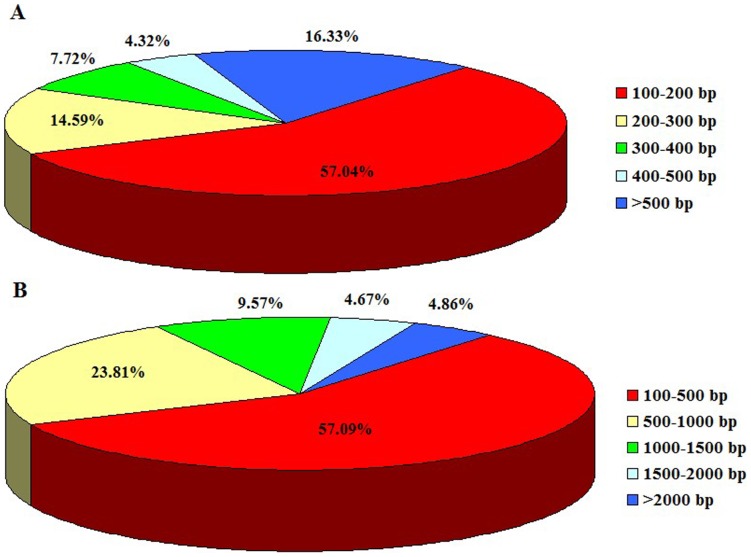
Overview of *Dastarcus helophoroides* transcriptome sequence assembly. (A) Size distribution of contigs obtained from clean reads. (B) Size distribution of unigenes generated by further assembly of contigs.

To obtain the *D. helophoroides* transcriptome, a cDNA library of adults was constructed. In total, 27,543,746 clean reads with an accumulated length of 2,478,937,140 bp were obtained after the removal of dirty reads from the raw reads using the filter_fq software (Table 1). More than 98% of the clean reads had quality scores higher than the Q20 level (an error probability of 1%). These high-quality clean reads were assembled *de novo* using the Trinity program, resulting in 86,032 contigs longer than 100 bp, with a mean length of 347 bp. Although the majority of the contigs were between 100 and 200 bp (57.04% of total), 14,046 (16.33%) were longer than 500 bp (Fig. 1A). Finally, the contigs were further assembled into 42,810 unigenes, including 8,469 clusters and 34,341 singletons. The mean length of unique transcripts was 683 bp, with 24,443 unigenes between 100 and 500 bp, 10,191 unigenes between 500 and 1000 bp, 4,099 unigenes between 1,000 and 1,500 bp, 1,998 unigenes between 1,500 and 2,000 bp, and 2,079 unigenes more than 2,000 bp (Fig. 1B).

**Table 1:** Overview of the transcriptome.

Total clean reads	27,543,746
Total clean nucleotides	2,478,937,140
Q20 percentage	98.54%
GC percentage	43.87%
Total number of contigs	86,032
Mean length of contigs	347
Total number of unigenes	42,810
Mean length of unigenes	683
N50 of unigenes	987
Distinct clusters	8,469
Distinct singletons	34,341

### Annotation of predicted proteins

**Figure 2:**
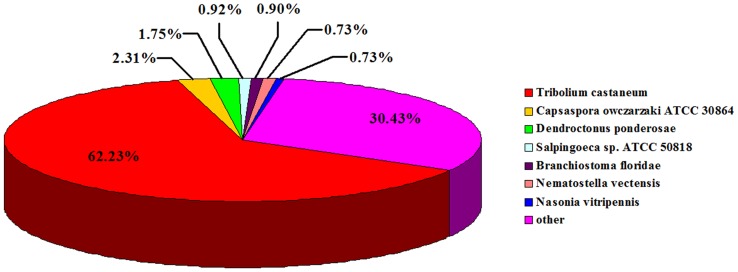
Species distribution of Blastx matches (with an E-value <10^−5^) to the unigenes against nr. Species distribution is shown as the percentage of total homologous sequences with an E-value of at least 10^−5^. We used the first hit of each sequence for analysis. Different colors represent different species.

All of the unigene sequences were annotated by searching the non-redundant (nr) NCBI protein database using BLASTX with a cutoff E-value of 10^−5^. A total of 30,103 distinct sequences (70.32% of unigenes) matched known genes (Table S1). The majority of sequences (62.23%) showed the highest homology with *Tribolium castaneum*, followed by *Capsapora owczarzaki* ATCC 30864 (2.31%) and *Dendroctonus ponderosae* (1.75%); however, species from other classes or organisms showed a lower similarity with *D. helophoroides.* The remaining 9,160 unigenes showed less than 0.73% similarity to other species, which consisted of 30.43% of our unique transcripts (Fig. 2).

### Functional annotation of unigenes

Alignments of Swiss-prot, Gene Ontology (GO) and Clusters of orthologous group (COG) databases were used to predict and classify potential functions of the unigenes. A total of 24,525 (57.29%) unigenes were annotated as 65,535 Swiss-Prot terms, each of which yielded a significant hit to one or more proteins.

**Figure 3:**
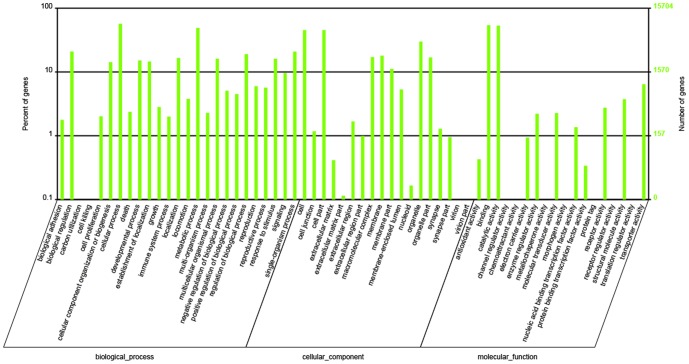
Gene Ontology annotation and classification of the *Dastarcus helophoroides* transcriptome. The results were summarized in three main categories: biological process, cellular component, and molecular function. The right y-axis indicates the number of genes in the category. The left y-axis indicates the percentage of a specific category of genes in that main category.

Among the 30,103 nr annotations, 15,704 were annotated as 98,975 GO terms, some of which participated in multiple GO terms. GO terms were used to classify the functions of the predicted *D. helophoroides* proteins. They were divided into three categories and 61 sub-categories (Fig. 3): biological process (25 sub-categories), cellular component (18 sub-categories), and molecular function (18 sub-categories). The majority of the GO terms consist of biological process (48,197; 48.69% of the total), followed by cellular component (30,729; 31.05%) and molecular function (20,049; 20.26%). The six major sub-categories were cellular process (8,941 GO terms) and metabolic process (7,659 GO terms) in the biological process, binding (8,465 GO terms) and catalytic activity (8,346 GO terms) in the molecular function, and cell (7,107 GO terms) and cell part (7,107 GO terms) in the cellular component, while the smallest groups were protein tag (two GO terms), receptor regulator activity (two GO terms), and chemoattractant activity (one GO term) in the molecular function and carbon utilization (only one GO term) in biological process.

Furthermore, all unigenes were searched against the COG database for functional prediction and classification. Because some of these unigenes received multiple COG annotations, 26,828 COG annotations were produced by 14,280 (33.36%) annotated unigenes. They were classified into 25 molecular families. The cluster of general function prediction (4,322; 16.11%) was the largest, followed by translation, ribosomal structure, and biogenesis (2,521; 9.39%) and posttranslational modification, protein turnover, and chaperones (2,002; 7.46%). The three smallest groups were RNA processing and modification (152; 0.57%), defense mechanisms (31; 0.12%), and nuclear structure (9; 0.03%).

### Metabolic pathway analysis of unigenes

All assembled unigenes were mapped to the Kyoto Encyclopedia of Genes and Genomes (KEGG). In total, 22,102 unigenes were located in 258 KEGG pathways. Metabolic pathways contained 3,848 unigenes (17.41%) and were significantly larger compared with other pathways, such as RNA transport (3.67%), regulation of the actin cytoskeleton (3.52%), and purine metabolism (3.50%) (Table S2).

### Genome size of D. helophoroides

**Figure 4:**
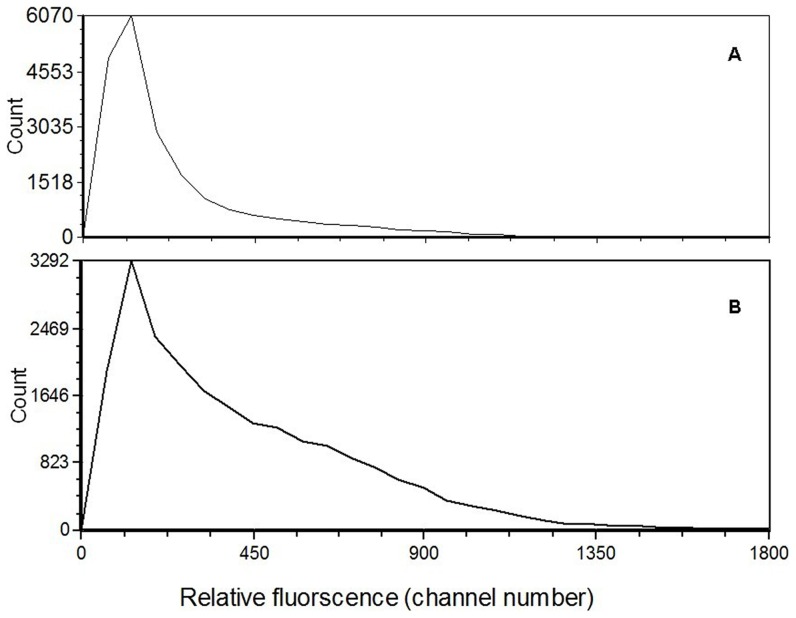
Relative DNA stainig in nuclei of *Tribolium castaneum* (A) and *Dastarcus helophoroides* (B). Relative red fluorescence of PI-stained nuclei shows a ratio of approximate 1.27:1.00 between T. castaneum (2C mean  = 122.0) and D. helophoroides (2C mean  = 96.0). PI staining time  = 4.8 h.

*D. helophoroides* nuclei had approximate 27% more DNA than *T. castaneum* (Fig. 4). Since genome sequencing papers have given genome size of 200 Mb for *T. castaneum*, it suggested that 2C in *D. helophoroides* is approximate 254 Mb.

### Data Deposition

Clean reads sequence data from *D. helophoroides* were deposited in the SRA database of the National Center for Biotechnology Information (NCBI, USA, http://www.ncbi.nlm.nih.gov/) with accession number SRP040468, while de novo assembly of sequence data in *D. helophoroides* were deposited in the Transcriptome Shotgun Assembly (TSA) database at DDBJ/EMBL/GenBank under the accession GBCX00000000. The version described in this paper is the first version, GBCX01000000.

## Discussion

*D. helophoroides* is the dominant natural enemy insect of many species of long-horned beetles and plays an important role in the biological control of these trunk borers. Despite its morphological, biological, and artificial reproduction research, molecular and sequence data of *D. helophoroides* are still limited. In this study, a reference transcriptome for *D. helophoroides* was sequenced and annotated using Illumina HiSeq 2000 sequencing technologies, and 2.48 Gb of transcriptome data were obtained and further assembled into 42,810 unigenes. Due to the lack of studies of *D. helophoroides* genes, to the best of our knowledge, this is the first study to obtain whole transcriptome information using RNA-seq in *D. helophoroides*. The genome size of 254 Mb for *D. helophoroides* was obtained by flow cytometry. And we estimated that the coverage of the *D. helophoroides* transcriptome was 9.8×. The number of unigenes (≥200 bp) obtained in this transcriptome analysis was approximately 2.1× the whole *D. helophoroides* genome. Our results provide the most extensive sequencing resource for further molecular studies on *D. helophoroides*.

Using transcriptome sequence analysis, it was found that *T. castaneum* shared the highest similarity with *D. helophoroides* in Blast annotation, whereas *D. ponderosae*, which also belonged to Coleoptera, showed a lower best match percentage. These results were likely due to the availability of more sequence resources of *T. castaneum* compared with *D. ponderosae* in the NCBI protein database because the number of protein sequences of *T. castaneum* and *D. ponderosae* was 27,489 and 16,252, respectively.

Currently, only 48 mRNA sequences are available (prior to September 24, 2013) in the NCBI database for *D. helophoroides*; obtaining more sequence information is thus a priority for researchers to perform gene function studies. In recent years, interest in the long lifespan and reproduction of *D. helophoroides* has increased in studies in China. However, the molecular mechanisms of development and reproduction remain unknown, and the main obstacle to further research is the limited amount of genetic information. The transcriptome of *D. helophoroides* provided abundant genetic information for further molecular study. Although a large number of potentially interesting genes were obtained from the transcriptome data, most of these genes were partial sequences of specific genes. Due to short size or poor alignment, some sequences were excluded from further analysis. Thus, to identify genes of interest using such data, particular attention should be paid to confirm that each unigene is unique. To resolve this problem, RACE technology is the preferred choice for future classification and to obtain the full length sequence of these genes.

In summary, whole transcriptome sequences of *D. helophoroides* were obtained using high-throughput sequencing, and the unigenes were annotated using five main databases. Taken together, these results will provide a solid foundation for further research of *D. helophoroides* at the molecular level.

## Conclusions

Using next-generation high-throughput sequencing, 42,810 unique sequences were obtained. Using a similarity search with known proteins, a total of 31,293 unigenes were identified to have BLAST hits with a cut-off E-value above 10^−5^. This is the first study to present transcriptome research on *D. helophoroides*. The transcriptome data provided a comprehensive sequence resource for future *D. helophoroides* study, thereby establishing an important public information platform for functional genomic studies in *D. helophoroides*.

## Materials and Methods

### Insect Samples

*D. helophoroides* were sampled from the Laboratory of Forestry Pests Biological Control, College of Forestry, Northwest Agriculture and Forestry University, Yangling in Shaanxi Province, People's Republic of China. Adults were reared in plastic boxes and fed an artificial diet that predominantly consisted of silkworm pupa powder, sugar, yolk, agar, and water. They were maintained in a temperature-controlled room at 22°C±1°C and 70±5% relative humidity and a photoperiod cycle of 16 h L/8 h D.

### RNA isolation and cDNA Library Preparation and Sequencing

Total RNA for transcriptome analysis was isolated from whole adults of *D. helophoroides* using Trizol reagent (Sangon Biotech, Shanghai, China). Four newly emerged (within two days of eclosion) adult individuals (two males and two females) were ground with liquid nitrogen into powder. The powder was quickly transferred into a 1.5 ml centrifuge tube and homogenized with 0.5 ml Trizol reagent. Total RNA extraction and purification was performed according to the manufacturer's instructions. Total RNA (A260/A280 = 2.057) was dissolved in 30 µl DEPC-treated H_2_O and stored at−80°C.

Total RNA was treated by DNase I to remove DNA. Next, magnetic beads with Oligo (dT) were used to isolate mRNA. The mRNA was mixed with fragmentation buffer and fragmented into short fragments. Next, the cDNA was synthesized using the mRNA fragments as templates. Short fragments were purified and resolved with EB buffer for end reparation and single nucleotide A (adenine) addition. Next, the short fragments were connected with adapters. Suitable fragments were selected for PCR amplification as templates. During the QC steps, Agilent 2100 Bioanalyzer and ABI StepOnePlus Real-Time PCR System were used to quantify and qualify the sample library. Finally, the library was sequenced using the Illumina HiSeq 2000 sequencer (Beijing Genomics Institute, BGI, Shenzhen, Guangdong, China). After sequencing, raw image data were transformed by base calling into sequence data, which were called raw data or raw reads and were stored in the fastq format.

### Transcriptome Assembly

Before performing bioinformatical analysis, the raw sequences were filtered to remove the low-quality reads. The filtration steps were as follows: 1) remove reads containing only the adaptor sequence; 2) remove reads containing unknown the nucleotide “N” over 5%; and 3) remove low quality reads (those with a ratio of bases with a quality value lower than 10 and occupying more than 20% of the entire read). The remaining clean reads were used for further analysis.

Transcriptome *de novo* assembly was performed using the short read assembling program Trinity (version release-20121005) [30]. The Trinity software first combined reads with a specific length of overlap to form longer fragments without N, forming contigs. Next, the reads were mapped back to contigs, and using paired-end reads, the software was able to detect contigs from the same transcript and the distances between these contigs. Next, Trinity connected these contigs to obtain consensus sequences that contained the least Ns and could not be extended on either end. Such sequences were defined as unigenes. Finally, Blastx alignments (E-value<10^−5^) between unigenes and sequences in protein databases, including the NR database, Swiss-Prot, KEGG, and COG, were performed to identify the sequence direction of unigenes. If the results of different databases conflicted, then a priority order of alignments from the NR, Swiss-Prot, KEGG, and COG databases was followed to determine the sequence direction. Unigenes that could not be aligned to any of the four databases were scanned using ESTScan [31], which produced a nucleotide sequence (5′–3′) direction and amino sequence of the predicted coding region. For unigenes with determined sequence directions, we identified their sequences from the 5′ to 3′ end and for those with undetermined directions and provided their sequence based on the assembly software (Table S3).

### Annotation of Functional Unigenes

Unigene annotation provides functional information. In our functional annotation, unigene sequences were first aligned using Blastx to the NR, Swiss-Prot, KEGG, and COG protein databases (E-value <10^−5^), which retrieved proteins with the highest sequence similarity to *D. helophoroides* unigenes in addition to their protein functional annotations. Homology searches were performed by querying the NCBI nr protein database using the Blastx algorithm (E-value <10^−5^) [32]. After NR annotation, the Blast2GO program [33] was used to obtain GO annotations, and the WEGO software [34] was used to perform GO functional classification of all unigenes to determine the distribution of gene functions at the macro level. KEGG is a database that analyzes the gene product during the metabolic process and related gene function in cellular processes. With the help of the KEGG database, we can further study the biological complex behaviors of genes, and using KEGG annotation, we can obtain pathway annotations for unigenes. After obtaining the KEGG pathway annotations, unigenes were aligned to the COG database to predict and classify potential functions based on known orthologous gene products. Every protein in COG is assumed to evolve from an ancestral protein, and the entire database is built on coding proteins with complete genomes as well as systematic evolutionary relationships among bacteria, algae, and eukaryotic organisms [35].

### Genome size of *D. helophoroides*

Samples were prepared for flow cytometry as described in Bennett *et al*. [36]. A single head of *D. helophoroides* or *T. castaneum* was placed into 1 ml Galbraith buffer, stroked 15 times with a JN92-IID Ultrasonic Cell Disruption System (work/rest  = 3 s/3 s) (Ningbo Jiangnan Instrument Factory, Zhejiang, China), and filtered through 20- µm nylon mesh.

Propidium iodide was added to each sample to a final concentration of 5 µg/ml, and the mixture co-stained in the dark at 4°C for a known duration of up to 24 h (usually 1–9 h). The mean fluorescence of stained nuclei in replicate samples was quantified, using a CyFlow Cube (Partec, Germany) flow cytometer with a laser tuned at 514 nm and 500 mW. Fluorescence at >615 nm was detected by a photomultiplier screened by a long pass filter.

## Supporting Information

Table S1 **Annotation of all of the unigenes.** 31,293 of the 42,810 unigenes were annotated using Blastx search in five public databases (NR, Swissprot, GO, COG, KEGG) with a cut-off E-value of 10^−5^.
(TXT)

Table S2 **KEGG analysis of 22,102 unigenes.**
(XLS)

Table S3 **Bioinformatics Protocol.**
(DOC)
