# Supplementary material for: Transcriptome Analysis of Dastarcus helophoroides (Coleoptera: Bothrideridae) Using Illumina HiSeq Sequencing
Source: PLoS One. 2014 Jun 30;9(6):e100673. doi: 10.1371/journal.pone.0100673 (PMC4076278; doi:10.1371/journal.pone.0100673)
Supplement: Table S3 — Bioinformatics Protocol. (DOC) [file pone.0100673.s003.doc]

Bioinformatics Protocol

**1. Pipeline of Experiments**

First, the total RNA is extracted and treated by DNase I. And then magnetic beads with Oligo (dT) are used to isolate mRNA (for eukaryotes). Mixed with the fragmentation buffer, the mRNA is fragmented into short fragments. Then cDNA is synthesized using the mRNA fragments as templates. Short fragments are purified and resolved with EB buffer for end reparation and single nucleotide A (adenine) addition. After that, the short fragments are connected with adapters. The suitable fragments are selected for the PCR amplification as templates. During the QC steps, Agilent 2100 Bioanaylzer and ABIStepOnePlus Real -Time PCR System are used in quantification and qualification of the sample library. At last, the library could be sequenced using Illumina HiSeq™ 2000 or other sequencer when necessary.

**2. Pipeline of Bioinformatics analysis**

**2.1 Output Statistics**

**Raw Sequence Data**

Image data output from sequencing machine is transformed by base calling into sequence data, which is called raw data or raw reads and stored in fastq format.

**Filter Raw Reads**

Raw reads produced from sequencing machines contain dirty reads which contain adapters, unknown or low quality bases. These data will negatively affect following bioinformatics analysis.

Therefore, dirty raw reads are discarded:

1. Remove reads with adaptors

2. Remove reads with unknown nucleotides larger than 5%

3. Remove low quality reads which the percentage of low quality bases (base quality≤10) is more than 20%

**Clean Reads**

The following analysis is based on clean reads, which are generated by filtering raw reads.

Total Raw Reads and Total clean Reads are actually total number of raw reads and total number of clean reads. Total clean Nucleotides is total number of clean nucleotides.

Q20 percentage is proportion of nucleotides with quality value larger than 20 in reads; N percentage is proportion of unknown nucleotides in clean reads. GC percentage is proportion of guanidine and cytosine nucleotides among total nucleotides.

**2.2 Assembly**

Transcriptome de novo assembly is carried out with short reads assembling program-Trinity. Trinity combines three independent software modules: Inchworm, Chrysalis, and Butterfly, applied sequentially to process large volumes of RNA-seq reads. Trinity partitions the sequence data into many individual de Bruijn graphs, each representing the transcriptional complexity at a given gene or locus, and then processes each graph independently to extract full-length splicing isoforms and to tease apart transcripts derived from paralogous genes. Briefly, the process works like so:

**Inchworm** Assembles the RNA-seq data into the unique sequences of transcripts, often generating full-length transcripts for a dominant isoform, but then reports just the unique portions of alternatively spliced transcripts.

**Chrysalis** Clusters the Inchworm Contigs into clusters and constructs complete de Bruijn graphs for each cluster. Each cluster represents the full transcriptonal complexity for a given gene (or sets of genes that share sequences in common). Chrysalis then partitions the full read set among these disjoint graphs.

**Butterfly** Then processes the individual graphs in parallel, tracing the paths that reads and pairs of reads take within the graph, ultimately reporting full-length transcripts for alternatively spliced isoforms, and teasing apart transcripts that corresponds to paralogous genes.

The result sequences of trinity are called Unigenes. When multiple samples from a same species are sequenced, Unigenes from each sample's assembly can be taken into further process of sequence splicing and redundancy removing with sequence clustering software to acquire non-redundant Unigenes as long as possible. Then do gene family clustering, the Unigenes will be divided to two class. One is clusters, which the prefix is CL and the cluster id is behind. In one cluster, there are several Unigenes which similarity between them is more than 70%. And the other is singletons, which the prefix is Unigene. In the final step, blastx alignment (evalue < 0.00001) between Unigenes and protein databases like NR, Swiss-Prot, KEGG and COG is performed, and the best aligning results are used to decide sequence direction of Unigenes. If results of different databases conflict with each other, a priority order of NR, Swiss-Prot, KEGG and COG should be followed when deciding sequence direction of Unigenes. When a Unigene happens to be unaligned to none of the above databases, a software named ESTScan [3] will be introduced to decide its sequence direction. For Unigenes with sequence directions, we provide their sequences from 5' end to 3' end; for those without any direction we provide their sequences from assembly software.

**2.3 Unigene Function Annotations**

Unigene annotation provides information of expression and functional annotation of Unigene. Information of functional annotation gives protein functional annotati on, COG functional annotation and Gene Ontology (GO) functional annotation of Unigenes.

Unigene sequences are firstly aligned by blastx to protein databases like NR, Swiss-Prot, KEGG and COG (e-value<0.00001), retrieving proteins with the highest sequence similarity with the given Unigenes along with their protein functional annotations, the results about this are including in the folder annotation.

KEGG database contains systematic analysis of inner-cell metabolic pathways and functions of gene products. It helps studying complicated biological behaviors of genes. With KEGG annotation we can get Pathway annotation of Unigenes.

COG is a database where orthologous gene products are classified. Every protein in COG is assumed to evolve from an ancestor protein, and the whole database is built on coding proteins with complete genome as well as system evolution relationships of bacteria, algae and eukaryotic creatures. Unigenes are aligned to COG database to predict and classify possible functions of Unigenes.

**2.4 Unigene GO Classification**

We can get GO functional annotation with NR annotation. Gene Ontology (GO) is an international standardized gene functional classification system which offers a dynamic updated controlled vocabulary and a strictly defined concept to comprehensively describe properties of genes and their products in any organism. GO has three ontologies: molecular function, cellular component and biological process. The basic unit of GO is GO-term. Every GO-term belongs to a type of ontology.

With NR annotation, we use Blast2GO program to get GO annotation of Unigenes. Blast2GO has been cited by other articles for more than 150 times and is a widely recognized GO annotation software. After getting GO annotation for every Unigene, we use WEGO software to do GO functional classification for all Unigenes and to understand the distribution of gene functions of the species from the macro level.

**2.5 Unigene Metabolic Pathway Analysis**

KEGG is a database that is able to analyze gene product during metabolism process and related gene function in the cellular processes. With the help of KEGG database, we can further study genes’ biological complex behaviors, and by KEGG annotation we can get pathway annotation for Unigenes.
